# Diagnosis and Treatment of Rotator Cuff Syndrome: A Narrative Review

**DOI:** 10.7759/cureus.105550

**Published:** 2026-03-20

**Authors:** Andres Eduardo Astorga Sosa, Maripaz Castro, Noilyn Nicolle Angulo Pichardo, Maria P Alfaro, Maria Jose Mora Navarro

**Affiliations:** 1 General Medicine, Universidad de Ciencias Médicas (UCIMED), San José, CRI; 2 General Medicine, Universidad Autónoma de Centro América (UACA), San José, CRI; 3 General Medicine, Universidad de Ciencias Médicas (UCIMED), San Jose, CRI

**Keywords:** conservative treatment, diagnostic imaging, rotator cuff tear, shoulder anatomy, shoulder pain

## Abstract

Rotator cuff disorders constitute one of the most frequent causes of shoulder pain and functional limitation, with a high prevalence in the adult population and a significant impact on quality of life. These conditions include a broad clinical spectrum ranging from tendinopathy and partial tears to full-thickness ruptures, and their clinical presentation does not always maintain a direct relationship with the magnitude of structural damage observed in imaging studies.

A thorough clinical assessment should remain the basis of the diagnosis, with imaging tests used wisely to support clinical findings rather than replace them. In addition, psychosocial factors, such as anxiety, depression, and unhelpful beliefs about pain, can considerably shape how patients experience and interpret their level of disability and should be considered when making therapeutic decisions.

​Conservative treatment is considered the initial strategy in most patients; it has been shown to have beneficial functional and quality-of-life outcomes, as compared to surgery, in the medium and long term, especially in degenerative or non-traumatic tears. Surgery should be reserved for selected cases, such as failure of non-surgical management or the presence of extensive lesions with a risk of irreversible progression, considering the high structural failure rates described.

​Overall, the management of rotator cuff syndrome must be individualized and centered on function, incorporating clinical evaluation, patient expectations, and the best available evidence, with the goal of optimizing clinical results and quality of life.

## Introduction and background

Rotator cuff syndrome is a clinical entity characterized by symptoms arising from the pathology of rotator cuff tendons, including pain, weakness, and functional limitation [1,2]. It encompasses a continuous spectrum of underlying structural abnormalities collectively referred to as rotator cuff disorders, ranging from tendinopathy and partial-thickness tears to full-thickness ruptures involving one or more tendons [1,3]. Within this spectrum, rotator cuff disorders represent the most common group of shoulder joint conditions, accounting for approximately 50% to 85% of cases [1]. It is estimated that after low back pain and knee pain, shoulder pain ranks third among the most common musculoskeletal consultations in primary care [1,2]. These conditions not only affect patients physically but are also associated with functional deterioration that compromises work capacity, recreational activities, and social interaction, often accompanied by psychological distress and reduced quality of life [1,4].

​Rotator cuff tears of traumatic origin show a higher prevalence in younger individuals, while degenerative origin are associated with progressive age-related tendon changes [5]. Imaging studies have shown that the occurrence of these lesions is associated with aging; up to 54% of asymptomatic patients older than 60 years present partial or complete rotator cuff tears [1,4]. In individuals over the age of 40, risk increases due to occupations requiring heavy lifting or repetitive overhead movements, as well as overuse in athletes exposed to constant microtrauma [4]. Despite the different therapeutic options available, clinical evolution is not always favorable, and it has been described that nearly 50% of patients treated in primary care continue to experience symptoms 6 months after the initial consultation [2].

​Given the range of clinical definitions and the broad range of available clinical interventions, the need for greater standardization in the approach to this pathology is evident. The purpose of this literature review is to integrate current scientific evidence on the diagnosis and treatment of rotator cuff syndrome to provide clarity regarding its management.

## Review

Methods

A comprehensive literature search, in both English and Spanish​​​​​, was performed in PubMed, Scopus, SciELO, and the Cochrane Library. The search included the term “rotator cuff” combined with keywords such as “tear,” “anatomy,” “pathophysiology,” “diagnosis,” and “therapeutic options.” Greater attention was given to studies published within the last five years in order to reflect the most recent evidence.

Articles were selected based on their clinical relevance and their contribution to understanding the anatomy, pathophysiology, diagnosis, and treatment of rotator cuff disorders. The review includes systematic reviews, meta-analyses, randomized controlled trials, observational studies, narrative reviews, diagnostic studies, translational research, and clinical practice guidelines from recognized scientific societies.

This work was developed as a structured narrative review rather than a formal systematic review. For this reason, no quantitative synthesis or standardized risk-of-bias assessment was conducted. The objective was to provide a clinically oriented and up-to-date synthesis of the available evidence.

Artificial intelligence tools, including Grammarly, were used as complementary resources during manuscript preparation to enhance language clarity, grammar, and the overall organization of the text. These tools were used solely to improve readability and linguistic quality. The authors retain full responsibility for the scientific content, interpretation, and final version of the manuscript.

​Anatomy and biomechanics of the rotator cuff

The rotator cuff is an integral musculotendinous structure composed of four main units: the subscapularis, supraspinatus, infraspinatus, and teres minor [6]. Together, they envelop the humeral head and allow wide mobility in multiple planes, making the shoulder the most mobile joint in the human body [7].

**Figure 1:**
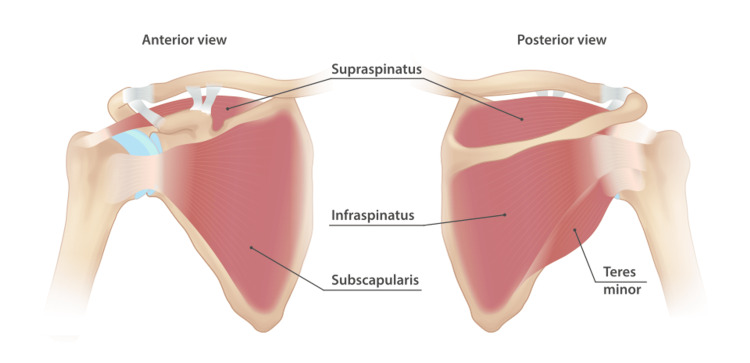
Anatomy of the rotator cuff muscles Illustration showing the rotator cuff musculature surrounding the humeral head, including the supraspinatus, infraspinatus, teres minor, and subscapularis muscles. Reproduced from InjuryMap, Rotator cuff muscles, Wikimedia Commons, licensed under Creative Commons Attribution-ShareAlike 4.0 (CC BY-SA 4.0) [8].

Individually, the subscapularis is the largest tendon that starts at the subscapular fossa, inserts into the lesser tuberosity, and functions as the primary internal rotator and anterior stabilizer [6]. The supraspinatus initiates the first 15° of arm abduction and inserts into the superior facet of the greater tuberosity [6]. The infraspinatus and teres minor begin in the greater tuberosity, specifically in the middle and inferior facets, respectively, and work coordinately in external rotation (Figure 1) [6].

The stability provided by the rotator cuff is essential for the proper function of the glenohumeral joint, particularly because the glenoid fossa is relatively small and represents only approximately one quarter of the size of the humeral head [9]. Consequently, joint stability relies largely on dynamic muscular mechanisms. One of the key stabilizing processes is the concavity-compression mechanism, in which the rotator cuff muscles compress the humeral head against the glenoid cavity during movement, maintaining joint congruency and stability [9].

This dynamic stabilization depends on the balance of two biomechanical force systems: the coronal force couple and the transverse force couple [7]. The coronal force couple represents the interaction between the deltoid muscle, which generates an upward force on the humeral head during arm elevation, and the subscapularis-infraspinatus-teres minor complex, which produces a downward and inward stabilizing force [7].

On the other hand, the transverse force couple reflects the anterior-posterior balance of forces across the glenohumeral joint [7]. In this system, the subscapularis provides an anteriorly directed force, while the infraspinatus and teres minor generate posteriorly directed forces. Together, these opposing forces contribute to the dynamic stabilization of the glenohumeral joint during rotational movements [7].

​Structural strength benefits from an intrinsic protective system known as the "suspension bridge," composed of the rotator cable and the rotator crescent [10]. The rotator cable is a thick group of fibers that redistributes mechanical stress away from the avascular zone of the rotator crescent, allowing the shoulder to function even in the existence of minor tears in that area [10].

​Regarding the properties of the rotator cuff tendon, it demonstrates an elastic response to deformation of up to 4% [7]. However, stresses exceeding 4% cause little damage to collagen fibers [7]. If deformation reaches between 8% and 10%, macroscopic failure occurs, while any deformation exceeding 10% inevitably results in complete tissue rupture, which is important to consider in the context of injuries [7].

Pathophysiology of the rotator cuff

​The pathophysiology of rotator cuff syndrome is currently understood as an active and multifactorial process, ranging from initial tendinopathy to full-thickness tears and terminal arthropathy [11]. This deterioration at the rotator cuff level has been explained through the interaction of extrinsic and intrinsic mechanisms [12].

Extrinsic impingement occurs when the tendon undergoes mechanical friction against structures such as the coracoacromial arch or osteophytes of the acromioclavicular joint [12]. On the other hand, the intrinsic mechanism indicates primary cuff degeneration due to aging, especially after 40 years of age, repetitive use, or poor vascularization [12]. It is essential to emphasize that microscopic alterations usually precede macroscopic rupture; in fact, tendons that appear intact to the naked eye may already show significant histopathological changes in patients with cuff disease [13].

​It is important to understand that the impact of the disease is not limited to the tendon but profoundly affects muscle quality, manifested by atrophy, fibrosis, and fatty infiltration [13]. This muscular degradation is significant because, once established, it is largely considered irreversible and predicts poor functional results even after successful surgical repair [14]. Recent research shows fibro-adipogenic progenitors (FAPs) as key cells in this degenerative process [14]. Although these stem cells initially play a protective role after injury, the persistence of pathological signals, such as transforming growth factor beta (TGF-β), drives them toward massive differentiation into adipocytes and fibroblasts [14]. This environment alters the "niche" of satellite cells, responsible for muscle regeneration, favoring proteolysis and programmed cell death of myocytes [14].

​Finally, the natural history of these lesions tends toward a vicious cycle of instability and further damage [12]. In chronic and massive tears, loss of the stabilizing function of the cuff allows superior migration of the humeral head [11]. This displacement alters joint mechanics and reduces the acromiohumeral interval, culminating in cuff tear arthropathy, a condition distinguished by severe glenohumeral arthritis and marked functional insufficiency [11].

​Clinical presentation

​Rotator cuff disorders may present with very nonspecific symptoms, but insidious-onset pain predominates, localized to the lateral aspect of the joint, which commonly worsens at night and causes significant sleep disturbances [11]. This pain is exacerbated when performing overhead activities, such as reaching high shelves or movements behind the back, required for personal hygiene and dressing tasks [11]. It is common for patients to manage the pain by taking analgesics and voluntarily avoiding painful movements, consulting a physician only when loss of strength or pain interferes with their daily life [7].

​To conduct a comprehensive clinical evaluation, clinical practice guidelines point out the importance of detailed medical history collection, precise measurement of the range of motion, and objective assessment of muscle strength [1,4,11]. It is necessary to identify red flags, which function as warning indicators of serious pathologies requiring urgent intervention, such as high-energy acute trauma, suspicion of infections, neoplasms, or acute neurological deficits. Likewise, identifying yellow flags, such as depression, anxiety, and catastrophic thinking, is associated with chronicity and prolonged disability [1].

​During physical examination, a painful arc of motion and objective weakness stand out, symptoms that tend to worsen at night or during overhead activities and posterior-reaching movements [9,11]. Although various individual diagnostic maneuvers exist, such as the Jobe test (empty can) or the Neer test [1,4,11]. The clinical practice guidelines of the American Academy of Orthopaedic Surgeons (AAOS) emphasize that combining multiple tests significantly increases diagnostic accuracy compared with the use of a single isolated test [4]. Finally, it is recommended to integrate the use of validated questionnaires, such as the PROMIS (Patient-Reported Outcomes Measurement Information System) system, to standardize quantification of functional status, mental health, and symptom intensity [1,15].

​A relevant finding in the recent literature is that the patient's reported disability does not directly correlate with the structural severity detected on imaging studies [15]. In effect, it has been demonstrated that levels of physical disability and perceived pain intensity are more deeply associated with signs of depression and anxiety than with traditional anatomic factors, such as tear size, degree of tendon retraction, or fatty muscle infiltration [16]. Individuals with major depressive episodes have a significantly higher probability of requesting specialized medical care and requesting surgical procedures as a means of resolving their symptoms [16]. The patient shows a loss of identity and autonomy, expressing frustration at being unable to fulfill personal, family, or work roles [17]. This clinical experience is aggravated by erroneous conceptions about the pathology, in which the individual interprets each episode of pain as a sign of imminent structural damage or of age-related "degeneration," strengthening a sense of physical vulnerability and promoting kinesiophobia [17]. Therefore, clinical presentation must be understood under a biopsychosocial model [15-17].

​Diagnosis

​The diagnosis of rotator cuff disorders is an integrative process that requires both clinical evaluation and rational use of imaging. The fundamental basis lies in a careful physical examination, especially in settings in which technological access is limited. In the initial evaluation, the main goal is to differentiate between inflammatory processes and structural tears. No single physical test is pathognomonic or sufficient on its own for a definitive diagnosis [18,19]. Current evidence suggests that we must be cautious when interpreting individual tests [9,18]. Rather than relying on a single sign, the literature recommends using combinations of tests to increase diagnostic effectiveness [19].​

Clinical evaluation of the rotator cuff lacks pathognomonic tests; therefore, the diagnostic odds ratio (DOR) is used to determine the utility of each maneuver [19]. The DOR is helpful because it shows how strongly a test result is linked to the presence of disease, and it combines sensitivity and specificity into one number, regardless of how common the disease is [19]. Within this context, the Jobe test stands out for its sensitivity (0.717), showing good ability to identify patients with rotator cuff tears, although it shows a modest DOR of 3.54 [19]. Similarly, the painful arc is still relevant in subacromial impingement syndrome, with a DOR of 2.81 [19]. However, this value reflects only moderate diagnostic effectiveness, consistent with evidence suggesting that no individual maneuver for subacromial impingement syndrome has sufficient discriminative capacity to confirm the condition in isolation [18,19].

To confirm a rotator cuff tear, maneuvers with high specificity are chosen to reliably “rule in” the diagnosis. Under this criterion, the External Rotation Lag Sign at 90° acts as the most precise maneuver, demonstrating near-perfect specificity (0.99) and the highest reported DOR (12.70) [19]. This performance markedly exceeds that of traditional maneuvers such as the Drop Arm (DOR 1.45) and the Lift-Off Test (DOR 3.00), which, despite their common use, did not reach diagnostic significance in meta-analyses using random-effects models. Due to the low quality of evidence, no single test allows these pathologies to be definitively excluded [4,18,19].

​When clinical evaluation is unclear, or surgery is being considered, imaging studies are necessary complements. Radiography is the starting point to rule out arthrosis or luxations. An acromiohumeral distance (AHD) less than 6-7 mm is a highly suggestive indirect sign of a massive tear and superior migration of the humeral head [7,11]. Ultrasound (US) is used because of its accuracy, cost, and safety [20]. In the hands of trained personnel, it may reach a sensitivity of 0.88 for full-thickness tears and 0.65 for partial-thickness tears. It is always recommended to corroborate with clinical findings and, in cases of diagnostic doubt, to request additional examinations [21].

Advanced imaging, such as magnetic resonance imaging (MRI) and magnetic resonance arthrography (MRA), should be considered for more specific findings. MRIs are useful in evaluating muscle tissue quality, allowing quantification of atrophy and fatty infiltration (Goutallier or Warner scales), which is critical for surgical prognosis. In full-thickness tears, its performance is excellent [20]. In comparison, MRAs represent the gold standard for partial tears, with a sensitivity of 83%, superior to conventional MRI and ultrasound in this specific category [20]. It is the tool of choice if additional intra-articular lesions or associated instability are suspected [22].​ CT is reserved for patients with contraindications to MRI or for complex osseous planning [15].

Additionally, arthroscopy is considered the reference diagnostic method because it allows direct and dynamic evaluation of the glenohumeral joint and subacromial space [4,23]. In clinical practice, it is used as a reference method to validate imaging studies and, in numerous cases, also acquires a therapeutic role by allowing treatment of the lesion during the same surgical act [23]. However, this technique is not used as a first-line diagnostic tool, due to considerations related to accessibility, costs, availability of resources, effectiveness of initial conservative management, and the stepwise diagnostic approach recommended in the evaluation of rotator cuff syndrome [4,23].

Treatment

Clinically, this group of pathologies is defined as rotator cuff-related shoulder pain, which encompasses conditions ranging from tendinopathy to partial and atraumatic tears [24,25]. Conservative management is established as the first-line intervention for rotator cuff lesions, as it allows mitigation of surgical risks and considerably reduces costs for the healthcare system [26,27]. This approach is considered the preferred therapeutic option in cases of degenerative tears or when tendon involvement is less than 50% of its total thickness [26,27].

​Rehabilitation is the foundation of the therapeutic strategy, with an emphasis on strengthening the fundamental shoulder musculature, improving joint stability, and optimizing proprioception [26]. Through individualized exercise plans, motor control-based programs are associated with greater reductions in pain and disability compared with other exercises, both in the short and medium to long term [28]. The most recent evidence suggests that the success of rehabilitation depends on three key components: patient education, progressive strengthening, and motor control [25].

Patient education is the first management strategy, aimed at lowering false beliefs and fears related to the pathology, thereby increasing self-efficacy and the patient’s understanding of their own condition [25]. Regarding physical activity, both strengthening through concentric contractions and eccentric training have shown positive results in muscle strength recovery and improvement of the tendon’s capacity to withstand load [25,26]. Likewise, motor control exercises seek to optimize muscle mobilization patterns and joint kinematics to prevent compression of subacromial soft tissues during movement [25].

In addition to exercise, the use of corticosteroids is one of the most common practices in orthopedic medicine, and it is considered effective by up to 96% of specialists for the relief of rotator cuff-related pain [24]. It is primarily characterized by providing rapid relief and a significant reduction in inflammation in early stages [26]. However, its benefit is usually temporary and, by itself, is not superior to physiotherapy [29]. There is growing controversy regarding its use due to the risk of cellular toxicity and the possibility that it may facilitate the progression of partial tears into full-thickness ruptures [24]. As alternatives, platelet-rich plasma (PRP), collagen, and hyaluronic acid have demonstrated effectiveness at reducing pain and improving functional strength in short-term follow-ups [24,26].

Conservative treatment is as effective as surgery in terms of functionality and quality of life after several years of follow-up, especially in patients older than 55 years with small and non-traumatic lesions [26,30]. Close medical follow-up with imaging studies is recommended, as non-surgical management does not always prevent muscle atrophy or fatty infiltration, processes that often occur asymptomatically and may transform an initially repairable lesion into an irreparable one [26,27].

**Figure 2:**
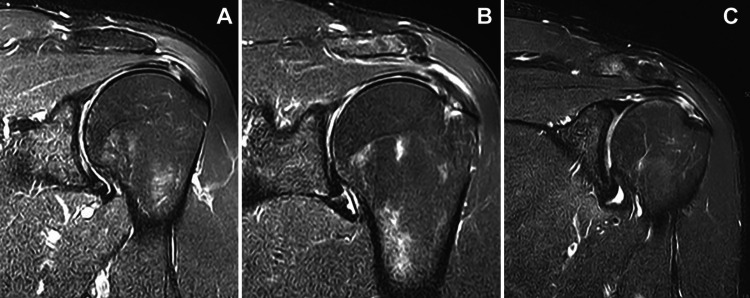
Ellman classification of partial-thickness rotator cuff tears (A) Grade I (<25%), (B) Grade II (25%–50%), and (C) Grade III (>50%) partial-thickness tears Reproduced from Park et al., Orthop J Sports Med, 2019, under the Creative Commons Attribution-NonCommercial-NoDerivatives 4.0 (CC BY-NC-ND 4.0) license [32].

In cases where conservative treatment fails to reach satisfactory results after a period of persistent pain and functional deterioration, or when tendon rupture exceeds 50% of its thickness (classified as a Grade III lesion or with a depth greater than 6 mm (Figure 2), surgical management becomes the main indication to restore anatomical integrity and prevent permanent functional deterioration [27]. The study "Effectiveness of open and arthroscopic rotator cuff repair (UKUFF)", a randomized trial conducted in 19 centers in the United Kingdom, found no difference in clinical effectiveness between open and arthroscopic repair at the 2-year follow-up [31]. This finding is largely attributed to a high re-tear rate of approximately 40% in both surgical groups, which ultimately equalized long-term clinical outcomes [31]. Additionally, the researchers noted that even when a re-tear occurred, many patients still experienced functional improvement, likely because the repair often healed partially, resulting in a smaller and less symptomatic defect as compared with the original lesion [31]. Although open surgery was associated with significantly shorter operative times, both techniques allowed marked improvement compared with patients’ baseline status [31].

To reduce this risk, the research "Arthroscopic rotator cuff repair combined with platelet-rich plasma products" illustrates the use of adjunctive biological therapies [33]. According to this meta-analysis of 21 controlled trials, intraoperative use of PRP substantially decreases the risk of repair failure, lowering the retear rate from 23.6% in control groups to 16.5% in patients treated with PRP [33]. PRP provides growth factors that improve the tendon’s natural healing process, additionally improving clinical outcomes such as the constant score and pain reduction (VAS) in the short, medium, and long term [32]. This benefit is especially notable in single-row (SR) repair techniques, where biomechanical strength is lower and PRP helps compensate for this vulnerability [33].

​It is important to consider the patient’s medical history when considering surgery. In individuals older than 55 years with small and non-traumatic supraspinatus tears, studies suggest that surgical treatment is not superior to physiotherapy in terms of functional gain or pain relief in the medium term [30]. However, excessive delay in surgery when it is necessary carries the risk that the lesion may become irreparable due to asymptomatic muscle atrophy and fatty infiltration, processes that modern surgical techniques still cannot reverse [27]. Therefore, the decision to operate must be personalized, prioritizing anatomical repair to restore function before permanent degenerative changes occur in the joint [30].

## Conclusions

Rotator cuff disorders are a frequent cause of shoulder pain and functional limitation, with significant clinical impact across multiple age groups. Current evidence indicates that the structural severity of the lesion does not always correlate with pain intensity or the degree of disability reported by the patient.

Conservative management is the most appropriate initial strategy in the majority of patients, especially in degenerative or partial lesions, offering functional outcomes comparable to surgery in the medium and long term. Rehabilitation based on education, progressive therapeutic exercise, and motor control constitutes the basis of treatment, while injections should be used selectively and with clear criteria. Surgical treatment should be considered or performed for cases in which conservative management has failed, larger tears are present, or lesions are at risk of irreversible progression. However, the high rates of structural failure after repair point to the need for appropriate patient selection and strategies targeted at optimizing tendon healing. Overall, the management of rotator cuff syndrome must be individualized, including clinical findings, patient expectations, and scientific evidence, prioritizing function and quality of life over isolated anatomical correction.
